# Treatment patterns, healthcare resource use, and costs associated with uncomplicated urinary tract infection among female patients in the United States

**DOI:** 10.1371/journal.pone.0277713

**Published:** 2022-11-21

**Authors:** Rena C. Moon, Alen Marijam, Fanny S. Mitrani-Gold, Daniel C. Gibbons, Alex Kartashov, Ning A. Rosenthal, Ashish V. Joshi

**Affiliations:** 1 Applied Research, PINC AI Applied Sciences, Premier Inc., Charlotte, NC, United States of America; 2 Value Evidence & Outcomes, GSK, Collegeville, PA, United States of America; 3 Epidemiology, GSK, Collegeville, PA, United States of America; 4 Value Evidence & Outcomes Real World Analytics, GSK, Brentford, Middlesex, United Kingdom; University of South Australia, AUSTRALIA

## Abstract

**Background:**

We evaluated associations between antibiotic prescription and healthcare resource use and costs (Part A), and between antibiotic switching and healthcare resource use, costs, and uncomplicated urinary tract infection recurrence (Part B) in female patients with uncomplicated urinary tract infection in the United States.

**Methods:**

This retrospective cohort study of linked Optum and Premier Healthcare Database data included female patients ≥12 years old with an uncomplicated urinary tract infection diagnosis (index date), who were prescribed antibiotics during an outpatient/emergency department visit between January 1, 2013 and December 31, 2018. In Part A, patients were stratified by antibiotic prescription appropriateness: appropriate and optimal (compliant with Infectious Diseases Society of America 2011 guidelines for drug class/treatment duration) versus inappropriate/suboptimal (inappropriate drug class/treatment duration per Infectious Diseases Society of America 2011 guidelines, and/or treatment failure). In Part B, patients were stratified by treatment pattern (antibiotic switch vs no antibiotic switch). Healthcare resource use and costs during index episode (within 28 days of index date) and 12-month follow-up were compared.

**Results:**

Of 5870 patients (mean age 44.5 years), 2762 (47.1%) had inappropriate/suboptimal prescriptions and 567 (9.7%) switched antibiotic. Inappropriate/suboptimal prescriptions were associated with higher healthcare resource use (mean number of ambulatory care and pharmacy claims [both p < 0.001]), and higher total mean cost (inpatient, outpatient/emergency department, ambulatory visits, and pharmacy costs) per patient ($2616) than appropriate and optimal prescriptions ($649; p < 0.001) (Part A). Antibiotic switching was associated with more pharmacy claims and higher total mean costs (p ≤ 0.01), and a higher incidence of recurrent uncomplicated urinary tract infection (18.9%) than no antibiotic switching (14.2%; p < 0.001) (Part B).

**Conclusions:**

Inappropriate/suboptimal prescriptions and antibiotic switching were associated with high costs, ambulatory care, and pharmacy claims, suggesting a need for improved uncomplicated urinary tract infection prescribing practices in the United States.

## Introduction

Urinary tract infections (UTIs) are a common infection in outpatient, emergency department (ED), and inpatient settings that are associated with a substantial burden on United States (US) healthcare systems [1–3]. UTI incidence in the US reportedly increased by 52% between 2008 and 2011 [4]: this was associated with increasing healthcare resource use (HRU) and costs, with total hospital admissions costs estimated at $2.8 billion in 2011 [4]. Prescription of appropriate antibiotics for UTI treatment is important for optimizing patient outcomes and limiting unnecessary HRU and costs.

Women are disproportionately affected by UTIs: it has been previously estimated that nearly 1 in 3 women will have at least one UTI episode requiring antibiotic treatment by 24 years of age [5], and their lifetime risk of UTI has been estimated to be 50–60% [6, 7]. UTIs in males, pregnant women, patients with comorbidities, immunosuppression, or an indwelling catheter, and in patients with an anatomical/functional abnormality of the urinary tract, are considered complicated. Uncomplicated UTIs (uUTIs) mostly occur in otherwise healthy women who are not pregnant, not immunosuppressed, and have no functional or structural urinary tract abnormalities [8].

According to the Infectious Disease Society of America (IDSA) 2011 Clinical Practice Guidelines [9], nitrofurantoin (NFT), trimethoprim-sulfamethoxazole (SXT), and fosfomycin trometamol are among the recommended first-line antibiotics for uUTI, with fluoroquinolones and β-lactams considered as alternate regimens wherever recommended first-line antibiotics cannot be used. While these guidelines have been widely disseminated among US clinicians, limited data on antibiotic prescriptions for uUTI (per IDSA 2011 prescribing guidelines) in clinical practice are available [10, 11]. Furthermore, we are unaware of any substantive data concerning the impact of inappropriate antibiotic prescriptions (i.e. prescriptions inconsistent with IDSA 2011 guidelines) on HRU and costs. A greater understanding of prescribing patterns could be beneficial in optimizing treatment practices and care among US patients with uUTI.

Our study used linked hospital discharge and claims data, including real-world prescription claims data, to evaluate prescribing patterns among US adult and adolescent female patients with uUTI. The aims of our study were twofold: first, we evaluated the association between appropriateness of antibiotic prescription and hospitalization risk, HRU, and costs (Part A); and secondly, we evaluated the relationship between antibiotic switching and HRU, costs, and UTI recurrence (Part B).

## Methods

### Study design

This was a retrospective cohort study conducted using data from the Optum and Premier Healthcare Database-linked database; all patients were previously discharged from a Premier Healthcare Database hospital. Identification of the study cohort and all inpatient-, outpatient-, and ED-related outcomes were assessed using Optum claims data.

### Patient sample

Female patients aged ≥12 years who were diagnosed with uUTI (index date) and prescribed oral antibiotics within ±5 days of an outpatient or ED visit between January 1, 2013 and December 31, 2018 were included in the study. UTI diagnosis was based on International Classification of Disease, Tenth Revision, Clinical Modification diagnosis codes (S1 Table in S1 File). Patients with recurrent uUTI (≥2 uUTI diagnoses [including index] within 6 months or ≥3 UTI diagnoses [including index] within 12 months) were included. To ensure sufficient claims data, eligible patients also needed to have continuous health plan enrollment with medical and pharmacy benefits ≥6 months prior to the index date and ≥12 months after the index date. Patients were excluded if they had complicated UTI, an acute or semi-acute infection (S2 Table in S1 File) ≤6 months before or <14 days after their visit, or an inpatient visit ≤3 months before or ≤2 days after the index date.

For Part A, eligible patients were stratified into two cohorts: those who received appropriate and optimal antibiotic prescriptions, and those who received inappropriate or suboptimal antibiotic prescriptions during the index uUTI episode. Appropriate antibiotic prescription was defined per IDSA 2011 guidelines for drug class and duration (S3 Table in S1 File) [9]. Inappropriate antibiotic prescription was defined as a prescription for an alternate antibiotic, two first-line antibiotics at the same time, or for an inappropriate treatment duration. Suboptimal antibiotic prescription, potentially due to wrong duration, wrong dose, or lack of compliance for antibiotics prescribed, was defined as evidence of treatment failure: treatment failure included receipt of intravenous antibiotics, a switch to a different antibiotic, or a primary diagnosis of UTI in an acute care setting within 28 days of the index date. Only patients with appropriate prescriptions and without evidence of suboptimal prescriptions were included in the appropriate and optimal cohort. Patients with either inappropriate or suboptimal prescriptions, or both, were included in the inappropriate/suboptimal cohort.

For Part B, eligible patients were stratified into two cohorts: antibiotic switch versus no antibiotic switch. The antibiotic switch cohort included patients who received ≥2 filled prescriptions of different antibiotics within 28 days following index date. The no antibiotic switch cohort included patients who received one filled antibiotic prescription during index uUTI episode.

Based on the US Title 45 Code of Federal Regulations (Part 46), Institutional Review Board approval for this study was not required because existing, deidentified hospital discharge data were used, and recorded information could not be identified directly or through identifiers linked to individuals. No informed consent of study participants was pursued due to the nature of the deidentified data.

### Study outcomes

One uUTI episode was defined as a 28-day period without evidence of treatment failure (i.e. no suboptimal antibiotic prescription). Where treatment failure was evident, the episode was extended for another 28 days from the date this occurred. In both Part A and Part B, the following study outcomes were assessed during the index uUTI episode and 12-month follow-up period: i) the percentage of patients with recurrent UTIs; ii) all-cause and UTI-related HRU, including the number of claims and the percentage of patients with these claims for inpatient, ED, and ambulatory care visits, and pharmacy services; iii) all-cause and UTI-related costs (inpatient, ED, and ambulatory visits, pharmacy services, and total cost); and iv) antibiotic treatment sequence, including drug name and percentage of antibiotics that were prescribed first, and drug name and percentage of antibiotics that were prescribed next (up to third treatment). UTI-related claims were associated with a principal or secondary UTI diagnosis. Costs were adjusted to 2019 US dollars using the medical care component of the Consumer Price Index.

### Statistical analysis

Descriptive statistics were used to describe patient characteristics and study outcomes. Continuous data were expressed as mean, standard deviation, median, and interquartile range; two sample comparisons were evaluated using a t-test or Wilcoxon Rank Sum test for continuous variables. Categorical data were expressed as counts and percentages of patients in each category; Chi-square or Fisher’s tests were used to test for statistical differences between comparison groups for categorical variables.

The independent effect of inappropriate or suboptimal antibiotic prescriptions on study outcomes was assessed using multivariable regression analyses, adjusting for age group, race, ethnicity, and Charlson Comorbidity Index (CCI) score category (0, 1–2, 3+). Multivariable logistic regression was used to assess the association between inappropriate or suboptimal antibiotic prescriptions and risks of inpatient, ED, or outpatient visits, and UTI recurrence; adjusted odds ratios (ORs) and 95% confidence intervals (CIs) were reported. We used generalized linear regression modeling to compare differences in costs between the two groups. For total cost during index episode, the model followed gamma loglink distribution and for total 12-month UTI-related and all-cause follow-up costs, the models followed inflated zero negative binomial distribution due to many zero values. All other costs were assessed only among those who incurred cost, and the models followed gamma loglink distribution. All adjusted means were estimated using recycled prediction method [12]: 95% CIs were estimated with bootstrapping method (1000 replicates with replacement). For all analyses, the statistical significance level was set to 0.05. All analyses were performed using SAS version 9.4 (Cary, NC, US).

## Results

### Baseline patient characteristics

A total of 5870 patients were included in the study (mean age 44.5 years; 76.6% White). For Part A, 2762 (47.1%) patients received an inappropriate (n = 1856) or suboptimal (n = 1255) antibiotic prescription (349 patients received both inappropriate and suboptimal prescriptions), and 3108 (52.9%) patients received an appropriate and optimal antibiotic prescription. Patients with an inappropriate or suboptimal antibiotic prescription were older than patients with an appropriate and optimal prescription (mean age 45.8 and 43.4 years, respectively); patients with an inappropriate or suboptimal prescription were also more likely to have Medicare and more likely to have a CCI score ≥1 versus patients with an appropriate or optimal prescription (p < 0.05 for all comparisons). For Part B, 567 (9.7%) patients switched antibiotics, while the remaining 5303 (90.3%) patients did not switch antibiotic. Patients who switched antibiotic were older than patients who did not switch antibiotic (mean age 46.9 and 44.2 years, respectively; p < 0.01). All baseline patient characteristics for Part A and Part B are detailed in Table 1.

**Table 1:** Baseline characteristics of patients stratified by appropriateness of antibiotic prescription.

Characteristic	Overall N = 5870	Part A			Part B		
		Inappropriate/suboptimal antibiotic prescription^a^ n = 2762 (47.1%)	Appropriate and optimal antibiotic prescription n = 3108 (52.9%)	p-value^a^	Antibiotic switch n = 567	No antibiotic switch n = 5303	p-value^b^
**Inappropriate antibiotic use, n (%)**	1856 (31.6)	1856 (67.2)	NA	NA	NA	NA	NA
**Suboptimal antibiotic use, n (%)**	1255 (21.4)	1255 (45.4)	NA	NA	NA	NA	NA
**Age group (years), n (%)**							
12–17	160 (2.7)	59 (2.1)	101 (3.2)	**< 0.001** ^**c**^	14 (2.5)	146 (2.8)	0.08
18–39	2890 (49.2)	1308 (47.4)	1582 (50.9)		252 (44.4)	2638 (49.7)	
40–59	1425 (24.3)	654 (23.7)	771 (24.8)		142 (25.0)	1283 (24.2)	
60–74	692 (11.8)	369 (13.4)	323 (10.4)		82 (14.5)	610 (11.5)	
75+	703 (12.0)	372 (13.5)	331 (10.6)		77 (13.6)	626 (11.8)	
**Age (years)**							
Mean (SD)	44.5 (20.0)	45.8 (20.6)	43.4 (19.3)	**< 0.001** ^**c**^	46.9 (20.0)	44.2 (20.0)	**< 0.01**
Median (IQR)	39.0 (30.0–58.0)	40.0 (30.0–61.0)	38.0 (30.0–55.0)		42.0 (32.0–61.0)	38.0 (30.0–58.0)	
**Race, n (%)**							
White	4498 (76.6)	2119 (76.7)	2379 (76.5)	0.26	441 (77.8)	4057 (76.5)	0.15
Black	473 (8.1)	236 (8.5)	237 (7.6)		34 (6.0)	439 (8.3)	
Other	899 (15.3)	407 (14.7)	492 (15.8)		92 (16.2)	807 (15.2)	
**Ethnicity, n (%)**							
Hispanic or Latino	545 (9.3)	280 (10.1)	265 (8.5)	0.08	53 (9.3)	492 (9.3)	0.88
Not Hispanic or Latino	3894 (66.3)	1826 (66.1)	2068 (66.5)		371 (65.4)	3523 (66.4)	
Unknown	1431 (24.4)	656 (23.8)	775 (24.9)		143 (25.2)	1288 (24.3)	
**Patients with Medicare coverage, n (%)**	1064 (18.1)	553 (20.0)	511 (16.4)	**< 0.001** ^**c**^	NA	NA	NA
**CCI score category, n (%)**							
0	5403 (92.0)	2512 (90.9)	2891 (93.0)	**0.01**	524 (92.4)	4879 (92.0)	0.81
1–2	456 (7.8)	243 (8.8)	213 (6.9)		43 (7.6)	413 (7.8)	
3+	11 (0.2)	7 (0.3)	4 (0.1)		0 (0.0)	11 (0.2)	

Abbreviations: CCI, Charlson Comorbidity Index; IQR, interquartile range; NA, not applicable; SD, standard deviation.

^a^Either inappropriate or suboptimal prescribing, values are not mutually exclusive.

^b^Comparisons made using Chi-square or Fisher’s tests.

^c^Statistically significant value (p < 0.05).

### All-cause and UTI-related HRU during index episode and 12-month follow-up

During the index uUTI episode, there were more ambulatory care (mean 8.0) and pharmacy (mean 3.3) claims per patient with inappropriate/suboptimal prescription than with appropriate and optimal prescription (means 6.3 [ambulatory care] and 2.6 [pharmacy], both p < 0.001) (Part A). Additionally, numbers of ambulatory care (mean 10.1) and pharmacy (mean 4.6) claims per patient, and the percentage of patients with UTI-related ED visits (17.3%) were higher in the antibiotic switch versus no antibiotic switch group (6.8, 2.8, and 12.0%, respectively; all p < 0.001) (Part B) (Table 2).

**Table 2:** Primary UTI-related HRU outcomes of uUTI outpatients stratified by inappropriate/suboptimal antibiotic prescription (Part A) and antibiotic switching (Part B) during the index episode.

	Overall N = 5870	Part A			Part B		
		Inappropriate/ suboptimal antibiotic prescription n = 2762	Appropriate and optimal antibiotic prescription n = 3108	p-value	Antibiotic switch n = 567	No antibiotic switch n = 5303	p-value
Inpatient visits, n (%)	2 (0.03)	2 (0.1%)	NA	NA	1 (0.2)	1 (0.02)	0.18
Number of inpatient visits, mean (SD)	1.0 (0.0)	1.0 (0)	NA	NA	1.0 (0.0)	1.0 (0.0)	NA
Inpatient length of stay, days, mean (SD)	1.5 (0.7)	1.5 (0.7)	NA	NA	1.0 (0.0)	2.0 (0.0)	NA
ED visits, n (%)	732 (12.5)	732 (26.5)	NA	NA	98 (17.3)	634 (12.0)	**< 0.001** ^**a**^
Number of ED claims, mean (SD)	5.6 (9.1)	5.6 (9.1)	NA	NA	6.1 (7.6)	5.5 (9.3)	0.53
Ambulatory care visits, n (%)	5856 (99.8)	2748 (99.5)	3108 (100)	**< 0.001** ^**a**^	565 (99.6)	5291 (99.8)	0.64
Number of ambulatory care claims, mean (SD)	7.1 (7.4)	8.0 (8.8)	6.3 (5.9)	**< 0.001** ^**a**^	10.1 (9.6)	6.8 (7.1)	**< 0.001** ^**a**^
Number of pharmacy claims, mean (SD)	2.9 (2.4)	3.3 (2.6)	2.6 (2.1)	**< 0.001** ^**a**^	4.6 (2.7)	2.8 (2.3)	**< 0.001** ^**a**^

Continuous variables were compared using Student’s t-test and categorical variables were compared using Chi-square or Fisher’s exact test. The denominator for the mean total cost is all patients. For patients who did not have follow-up visits, the cost was set to 0. For all other cost variables, the cost was set to missing if the patient did not incur any cost.

Abbreviations: ED, emergency department; HRU, healthcare resource use; NA, not applicable; SD, standard deviation; UTI, urinary tract infection; uUTI, uncomplicated urinary tract infection.

^a^Statistically significant value (p < 0.05).

During the 12-month follow-up period, 861 (14.7%) patients had a recurrent UTI. A higher percentage of patients had recurrent uUTI in the antibiotic switch group versus the no antibiotic switch group (18.9% vs 14.2%; p < 0.001), and there was a slightly higher mean number of uUTI episodes per patient in patients who switched antibiotic than in those who did not switch antibiotic (1.4 vs 1.3, respectively; p < 0.001) (Part B) (Table 3). Compared with patients who did not have recurrent UTI, patients with recurrent UTI were older (mean age 43.4 vs 50.9 years) and were more likely to have Medicare coverage (16.3% vs 29.0%), a higher mean CCI score (0.1 vs 0.2), and an inappropriate (31.3% vs 33.6%) or suboptimal antibiotic prescription (21.0% vs 23.3%) (data not shown; all p < 0.001). There were more patients with UTI-related ED visits with inappropriate or suboptimal prescription (9.7%) than with an appropriate and optimal prescription (6.5%; p < 0.001) (Part A). Patients in the antibiotic switch group were more likely to have UTI-related ED visits (10.8%), ambulatory care visits (27.2%), and pharmacy claims (23.6%) than patients in the no antibiotic switch group (7.7%, 21.9%, and 19.0%, respectively; all p ≤ 0.01), and patients who switched antibiotic had a higher mean number of pharmacy claims than those who did not switch antibiotic (2.9 vs 2.2, respectively; p < 0.001). Additionally, patients in the antibiotic switch group had more all-cause inpatient (17.5%) and ED visits (28.9%) than those in the no antibiotic switch group (13.2% and 23.8%, respectively; both p < 0.01) (Part B) (Table 3).

**Table 3:** Overall, UTI-related, and all-cause HRU outcomes of uUTI outpatients stratified by inappropriate/suboptimal antibiotic prescription (Part A) and antibiotic switching (Part B) during 12-month follow-up.

	Overall N = 5870	Part A			Part B		
		Inappropriate/suboptimal antibiotic prescription n = 2762	Appropriate and optimal antibiotic prescription n = 3108	p-value	Antibiotic switch n = 567	No antibiotic switch n = 5303	p-value
**Overall outcomes of uUTI during the 12-month follow-up**							
28-day risk of UTI-related hospitalization, n (%)	2 (0.03)	2 (0.1%)	NA	NA	1 (0.2)	1 (0.02)	0.18
Number of uUTI episodes, mean (SD)	1.3 (0.7)	1.3 (0.7)	1.3 (0.7)	0.10	1.4 (0.9)	1.3 (0.7)	**< 0.001** ^**a**^
Recurrent uUTI, n (%)	861 (14.7)	433 (15.7)	428 (13.8)	**< 0.05**	107 (18.9)	754 (14.2)	**< 0.001** ^**a**^
**UTI-related HRU outcomes during 12-month follow-up**							
Inpatient visits, n (%)	33 (0.6)	21 (0.8)	12 (0.4)	0.06	2 (0.4)	31 (0.6)	0.77
Number of inpatient claims, mean (SD)	2.4 (1.9)	2.5 (1.8)	2.3 (2.0)	0.84	4.0 (1.4)	2.3 (1.9)	0.22
Inpatient length of stay, days, mean (SD)	23.2 (29.0)	19.4 (26.4)	29.9 (33.3)	0.33	14.5 (7.8)	23.8 (29.9)	0.67
ED visits, n (%)	469 (8.0)	267 (9.7)	202 (6.5)	**< 0.001** ^**a**^	61 (10.8)	408 (7.7)	**0.01** ^**a**^
Number of ED claims, mean (SD)	20.0 (32.6)	18.9 (26.4)	21.4 (39.4)	0.41	14.9 (17.9)	20.7 (34.2)	0.19
Ambulatory care visits, n (%)	1313 (22.4)	631 (22.8)	682 (21.9)	0.41	154 (27.2)	1159 (21.9)	**< 0.01** ^**a**^
Number of ambulatory care claims, mean (SD)	48.2 (55.3)	52.6 (61.3)	44.1 (48.8)	**< 0.01**	55.4 (52.7)	47.3 (55.6)	0.08
Pharmacy claims, n (%)	1141 (19.4)	546 (19.8)	595 (19.1)	0.55	134 (23.6)	1007 (19.0)	**< 0.01** ^**a**^
Number of pharmacy claims, mean (SD)	2.3 (1.8)	2.3 (1.9)	2.2 (1.7)	0.52	2.9 (2.5)	2.2 (1.7)	**< 0.001** ^**a**^
**All-cause HRU outcomes during 12-month follow-up**							
Inpatient visits, n (%)	799 (13.6)	422 (15.3)	377 (12.1)	**0.001** ^**a**^	99 (17.5)	700 (13.2)	**< 0.01** ^**a**^
Number of inpatient claims, mean (SD)	1.6 (1.2)	1.6 (1.2)	1.6 (1.2)	0.54	1.5 (1.0)	1.6 (1.2)	0.31
Inpatient length of stay, days, mean (SD)	11.8 (20.9)	12.5 (22.0)	11.0 (19.6)	0.32	8.8 (13.0)	12.2 (21.8)	0.13
ED visits, n (%)	1424 (24.3)	781 (28.3)	643 (20.7)	**< 0.001** ^**a**^	164 (28.9)	1260 (23.8)	**< 0.01** ^**a**^
Number of ED claims, mean (SD)	13.2 (23.5)	13.2 (20.6)	13.3 (26.5)	0.94	11.6 (14.6)	13.4 (24.4)	0.35
Ambulatory care visits, n (%)	4524 (77.1)	2084 (75.5)	2440 (78.5)	**< 0.01** ^**a**^	430 (75.8)	4094 (77.2)	0.46
Number of ambulatory care claims, mean (SD)	24.6 (29.2)	25.5 (30.9)	23.9 (27.8)	0.06	28.2 (27.7)	24.3 (29.4)	**< 0.01** ^**a**^
Pharmacy claims, n (%)	5263 (89.7)	2501 (90.6)	2762 (88.9)	**0.03** ^**a**^	537 (94.7)	4726 (89.1)	**< 0.001** ^**a**^
Number of pharmacy claims, mean (SD)	18.3 (21.6)	19.4 (21.8)	17.4 (21.4)	**< 0.001** ^**a**^	20.7 (21.4)	18.1 (21.6)	**< 0.01** ^**a**^

Continuous variables were compared using Student’s t-test and categorical variables were compared using Chi-square or Fisher’s exact test. The denominator for the mean total cost is all patients. For patients who did not have follow-up visits, the cost was set to 0. For all other cost variables, the cost was set to missing if the patient did not incur any cost.

Abbreviations: ED, emergency department; HRU, healthcare resource use; NA, not applicable; SD, standard deviation; UTI, urinary tract infection; uUTI, uncomplicated urinary tract infection.

^a^Statistically significant value (p < 0.05).

After adjusting for patient characteristics, the likelihood of having a UTI-related ED visit during 12-month follow-up was higher in patients who received inappropriate or suboptimal prescriptions than in those who received appropriate and optimal prescriptions (OR 1.40; 95% CI 1.15–1.71). Within 12 months of the index episode, and after adjustment for age group, race, ethnicity, and CCI score, the odds of having an all-cause inpatient visit (OR 1.20; 95% CI 1.02–1.41) and the odds of having an all-cause ED visit (OR 1.45; 95% CI 1.28–1.64) were higher in the inappropriate and suboptimal cohort compared with the appropriate and optimal cohort.

### All-cause and UTI-related costs during index episode and 12-month follow-up

During the index episode, UTI-related mean total cost per patient was higher with inappropriate or suboptimal prescribing ($2616) than with appropriate and optimal prescribing ($649; p < 0.001) (Part A), and there were higher UTI-related mean total costs per patient in the antibiotic switch group ($2186) than in the no antibiotic switch group ($1509; p = 0.01) (Part B) (Table 4).

**Table 4:** Primary UTI-related cost outcomes of uUTI outpatients stratified by inappropriate/suboptimal antibiotic prescription (Part A) and antibiotic switching (Part B) during the index episode.

	Overall N = 5870	Part A			Part B		
		Inappropriate/ suboptimal antibiotic prescription n = 2762	Appropriate and optimal antibiotic prescription n = 3108	p-value	Antibiotic switch n = 567	No antibiotic switch n = 5303	p-value
Mean (SD) outpatient ED visit costs, $	3412 (7610)	3412 (7610)	NA	NA	4861 (5631)	3188 (7852)	**0.04** ^**a**^
Mean (SD) ambulatory care visit costs, $	1023 (4848)	1564 (6632)	546 (2218)	**< 0.001** ^**a**^	1155 (3771)	1009 (4949)	0.50
Mean (SD) pharmacy costs, $	126 (985)	150 (1404)	104 (278)	0.07	179 (324)	120 (1031)	0.17
Mean (SD) total costs during index uUTI episode, $	1574 (6072)	2616 (8405)	649 (2246)	**< 0.001** ^**a**^	2186 (5336)	1509 (6142)	**0.01** ^**a**^

Continuous variables were compared using Student’s t-test and categorical variables were compared using Chi-square or Fisher’s exact test. The denominator for the mean total cost is all patients. For patients who did not have follow-up visits, the cost was set to 0. For all other cost variables, the cost was set to missing if the patient did not incur any cost.

Abbreviations: ED, emergency department; NA, not applicable; SD, standard deviation; UTI, urinary tract infection; uUTI, uncomplicated urinary tract infection.

^a^Statistically significant value (p < 0.05).

During the 12-month follow-up period, mean total UTI-related costs per patient ($5048) and total all-cause costs ($12,539) were higher with inappropriate or suboptimal prescribing than with appropriate and optimal prescribing ($3633 and $10,510, respectively; both p = 0.01) (Part A). Mean pharmacy costs ($74) and UTI-related ($5502) and all-cause ($12,960) costs were higher for the antibiotic switch group than the no antibiotic switch group ($44, $4171, and $11,305, respectively; p ≤ 0.23) (Part B) (Table 5).

**Table 5:** Primary UTI-related and all-cause costs of uUTI outpatients stratified by inappropriate/suboptimal antibiotic prescription (Part A) and antibiotic switching (Part B) during 12-month follow-up.

	Overall N = 5870	Part A			Part B		
		Inappropriate/suboptimal antibiotic prescription n = 2762	Appropriate and optimal antibiotic prescription n = 3108	p-value	Antibiotic switch n = 567	No antibiotic switch n = 5303	p-value
**UTI-related costs during 12-month follow-up, $**							
Mean (SD) ambulatory care visit costs	12,583 (31,163)	13,688 (27,984	11,559 (33,836)	0.22	13,996 (28,973)	12,395 (31,450)	0.55
Mean (SD) pharmacy costs	48 (89)	49 (96)	46 (81)	0.55	74 (136)	44 (80)	**< 0.001** ^**a**^
Mean (SD) total UTI-related costs	4299 (21,141)	5048 (21,916)	3633 (20,409)	**0.01** ^**a**^	5502 (23,287)	4171 (20,897)	0.15
**All-cause costs during 12-month follow-up, $**							
Mean (SD) inpatient visit costs	35,658 (44,986)	35,042 (38,322)	36,343 (51,436)	0.69	30,878 (36,840)	36,326 (45,993)	0.27
Mean (SD) outpatient ED visit costs	12,217 (21,734)	12,361 (19,839)	12,043 (23,854)	0.78	12,239 (19,608)	12,215 (22,003)	0.99
vMean (SD) ambulatory care visit costs	3566 (9451)	3657 (7970)	3488 (10,553)	0.55	3856 (6677)	3536 (9696)	0.50
Mean (SD) pharmacy costs	1194 (3325)	1244 (2764)	1150 (3762)	0.31	1454 (3659)	1165 (3284)	0.06
Mean (SD) total all-cause costs	11,465 (31,385)	12,539 (29,936)	10,510 (32,594)	**0.01** ^**a**^	12,960 (29,900)	11,305 (31,538)	0.23

Continuous variables were compared using Student’s t-test and categorical variables were compared using Chi-square or Fisher’s exact test. The denominator for the mean total cost is all patients. For patients who did not have follow-up visits, the cost was set to 0. For all other cost variables, the cost was set to missing if the patient did not incur any cost.

Abbreviations: ED, emergency department; SD, standard deviation; UTI, urinary tract infection; uUTI, uncomplicated urinary tract infection.

^a^Statistically significant value (p < 0.05).

**Fig 1:**
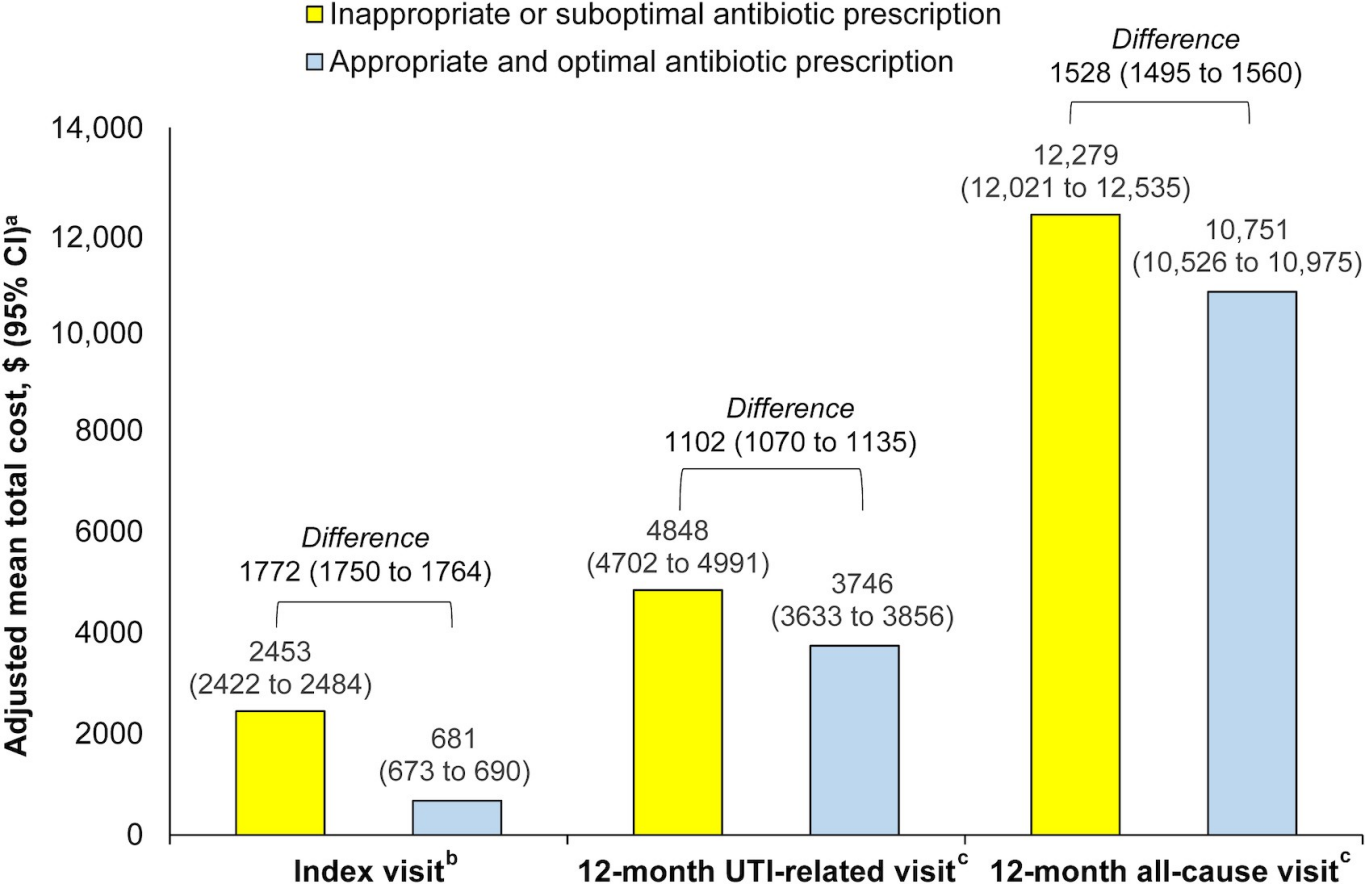
Total 12-month UTI-related and all-cause visit costs (adjusted), stratified by appropriateness of antibiotic prescription at index and during follow-up. Abbreviations: CCI, Charlson Comorbidity Index; CI, confidence interval; ED, emergency department; US, United States; UTI, urinary tract infection. ^**a**^All cost calculations were adjusted to 2019 US dollars based on Consumer Price Index for all urban consumers for hospital and related services. All models were adjusted for age group (reference: 18–39 years), race/ethnicity (reference: White and non-Hispanic), and CCI (reference: 0 CCI). ^**b**^Inpatient and ED visit costs during the index UTI episode were not modeled because, by definition, the appropriate and optimal antibiotic prescription group did not incur any costs. ^**c**^For total 12-month UTI-related and all-cause visit costs, the models followed inflated zero negative binomial distribution (due to many zero costs in the outcome variable) and used recycled prediction modeling.

Adjusted total costs per patient were higher for the inappropriate or suboptimal prescription group than for the appropriate and optimal prescription group at index visit (by $1772), and in terms of both UTI-related (by $1102) and all-cause (by $1528) costs at 12-month follow-up (Fig 1). All other adjusted costs at index and 12-month follow up (UTI-related and all-cause) are shown in S4 Table in S1 File.

### Antibiotic treatment sequence

**Fig 2:**
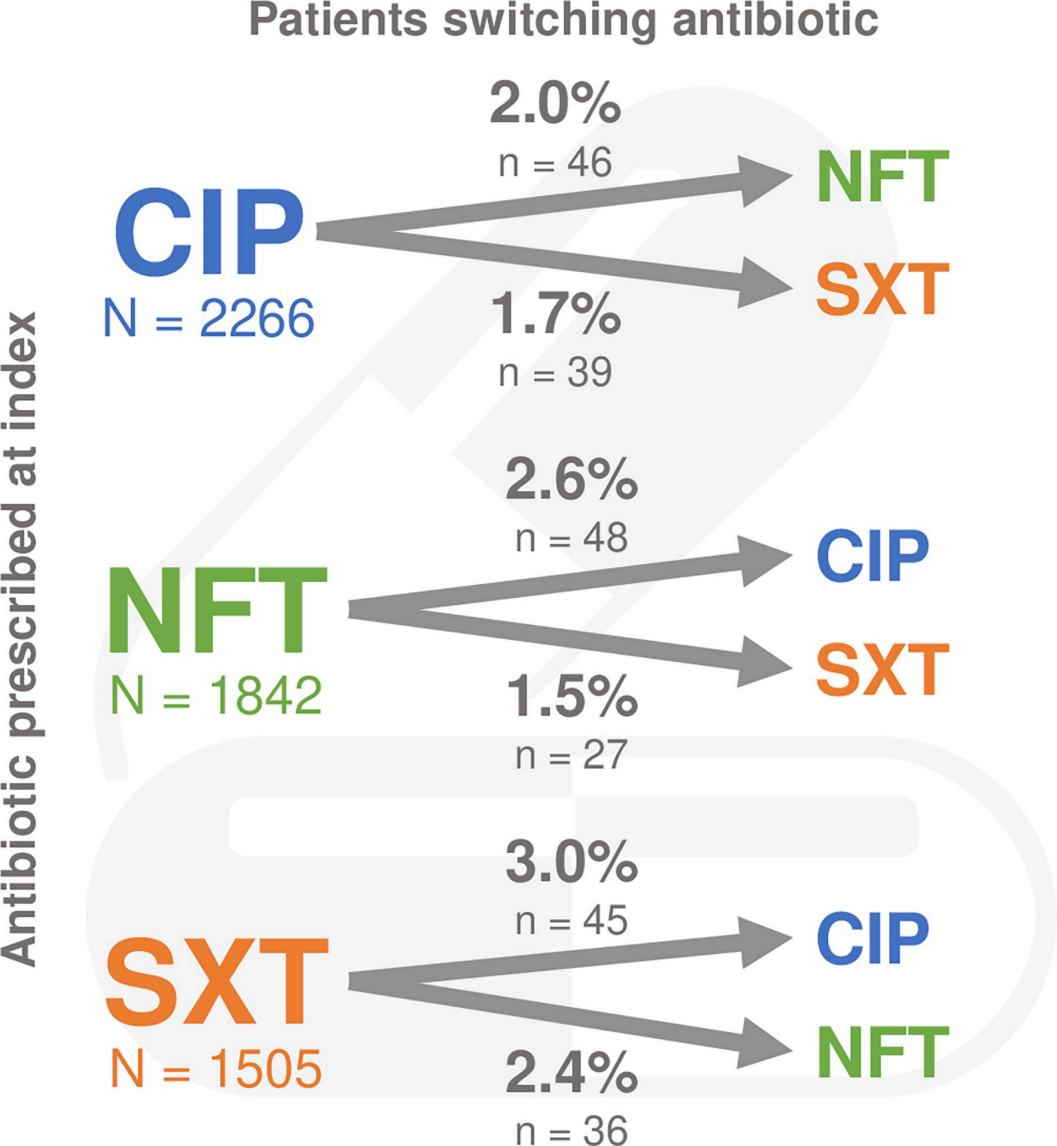
Antibiotic switching patterns at index date. Abbreviations: CIP, ciprofloxacin; NFT, nitrofurantoin; SXT, trimethoprim-sulfamethoxazole.

Among all patients (N = 5870), the most prescribed first-line antibiotics at index were ciprofloxacin (CIP; n = 2266 [38.6%]), NFT (n = 1842 [31.4%]), and SXT (n = 1505 [25.6%]). Of patients prescribed CIP, 2.0% switched to NFT and 1.7% switched to SXT. Of patients prescribed NFT, 2.6% switched to CIP and 1.5% switched to SXT. Of patients prescribed SXT, 3.0% switched to CIP and 2.4% switched to NFT (Fig 2). All first-line antibiotics and corresponding treatment patterns are detailed in S5 Table in S1 File.

## Discussion

Our results suggest that an inappropriate or suboptimal antibiotic prescription for uUTI is common in the US, and that this practice is associated with higher HRU and costs as well as an increased risk of recurrent UTI infection. Furthermore, these data suggest that antibiotic switching (i.e. the practice of changing the initially prescribed antibiotic within 28 days after uUTI diagnosis) is also significantly associated with higher HRU and an increased risk of UTI recurrence, compared with not switching antibiotics.

Inappropriate or suboptimal antibiotic prescriptions were observed in nearly half of all outpatient visits for uUTI and were associated with higher costs and mean numbers of ambulatory care and pharmacy claims during the index uUTI episode, compared with appropriate and optimal antibiotic prescriptions for uUTI. Patients with inappropriate or suboptimal antibiotic prescriptions were also more likely to visit the ED due to UTI within 12 months of the index episode than patients with appropriate and optimal antibiotic prescriptions. Despite the availability of well-established treatment guidelines [9], prescribing practices have shown to vary and are not always consistent with the guidelines. A chart review study involving 128 adult patients with uUTI from an internal medicine clinic during 2012–2013 indicated that only 64.1% of patients were prescribed appropriate first- or second-line antibiotics per IDSA 2011 guidelines (fewer patients had an appropriate treatment duration than a choice of medication) [11]. In a large commercially insured sample of 654,432 nonpregnant women aged 18–44 years in the US, collected between January 1, 2009 and December 31, 2013, Durkin et al. found that fluoroquinolone was the most commonly prescribed antibiotic class both before and after the release of the IDSA 2011 guidelines (45% and 42%, respectively), and >75% of prescriptions did not follow the recommended treatment durations [10]. This suggested a lack of awareness of guideline recommendations among physicians that subsequently contributed to inappropriate antibiotic prescription patterns [10]. Such a trend of inappropriate antibiotic prescribing was also observable in our study even 7 years after the issue of the IDSA 2011 treatment guidelines, suggesting an ongoing unmet need to raise awareness of UTI prescribing guidelines among physicians in the US.

Antimicrobial resistance to routinely prescribed antibiotics results in higher costs, health consequences, and challenges in the treatment of UTIs [4, 13], which can lead to patients switching antibiotic treatment. Additionally, if a patient’s symptoms do not resolve following prescription of empiric therapy per IDSA guidelines [9], antibiotic switching can take place. Awareness of antimicrobial resistance and established guidelines for appropriate antibiotic prescribing practices are required in the empirical treatment of UTIs [14].

This study used real-world data, providing insight into HRU and costs associated with inappropriate and/or suboptimal antibiotic prescriptions, and the impact of antibiotic switching. In addition, these data have highlighted an unmet need for training to optimize prescribing practices for uUTI per IDSA guidelines.

There are several study limitations. uUTI identification was based on diagnosis codes and a series of rule-out conditions; therefore, uUTI misclassification may have occurred. If a complicated UTI was included in a cohort it may have inflated the calculations for associated HRU and costs; however, we expect minimal effect on comparisons since misclassification would have been non-differential across different comparison groups. Furthermore, the presence of a claim for a filled prescription does not indicate whether the medication was taken by the patient or whether used as prescribed. This was a retrospective observational study, so we were only able to report associations and cannot comment directly on potential causality. There were insufficient data on the results of urine microscopy, culture, and sensitivity to ascertain the reasons for antibiotic switching (e.g. due to antibiotic resistance or re-infection with another uropathogen), and we were only able to assess comorbidities reported during the index visit and/or visits to healthcare settings during the 6 months prior to index visit. If a patient did not have a clinical visit during the assessment period, comorbidities may have been underestimated, although any underestimation would be non-differential across different comparison groups. The study only used a subset of the Optum database and relied on linked data to follow-up health outcomes post-admission. Due to this, the results of this study may not be generalizable outside of the patient sample analyzed from the US, and so additional research into this field incorporating healthcare claims from a range of databases would be beneficial in expanding the potential reach of the data.

## Conclusions

Using real-world data, we observed a positive association between inappropriate or suboptimal antibiotic prescription and antibiotic switching with higher HRU and costs, compared with no antibiotic switching and appropriate and optimal antibiotic prescriptions among female outpatients with uUTI. Patients who switched antibiotic were also more likely to experience a recurrent UTI. Our findings have subsequent implications for the need to improve prescribing practices and prescriber education for uUTI, as means of optimizing patient care in the US.

## Supporting information

S1 File (DOCX)
