# Supplementary material for: Treatment patterns, healthcare resource use, and costs associated with uncomplicated urinary tract infection among female patients in the United States
Source: PLoS One. 2022 Nov 21;17(11):e0277713. doi: 10.1371/journal.pone.0277713 (PMC9678295; doi:10.1371/journal.pone.0277713)
Supplement: S1 File — (DOCX) [file pone.0277713.s001.docx]

**Supporting information**

**S1 Table. ICD diagnosis codes and descriptions for UTI.**

| **Diagnosis code** | **ICD-version** | **Description** |
| --- | --- | --- |
| 595.0 | ICD-9 | Acute cystitis |
| 595.9 | ICD-9 | Cystitis, unspecified |
| 599.0 | ICD-9 | Urinary tract infection, site not specified |
| N30.00 | ICD-10 | Acute cystitis without hematuria |
| N30.01 | ICD-10 | Acute cystitis with hematuria |
| N30.90 | ICD-10 | Cystitis, unspecified without hematuria |
| N30.91 | ICD-10 | Cystitis, unspecified with hematuria |
| N39.0 | ICD-10 | Urinary tract infection, site not specified |

Abbreviations: ICD-9, International Classification of Disease, 9^th^ Revision; ICD-10, International Classification of Disease,10^th^ Revision; UTI, urinary tract infection.

**S2 Table. ICD diagnosis codes and descriptions for acute and semi-acute infections.**

| **ICD-9 code** | **ICD-10 code** | **Description** |
| --- | --- | --- |
| 460–466, 473 | J00, J01.00, J01.10, J01.20, J01.30, J01.40, J01.90, J02.9, J03.90, J04.0, J05.0, J04.10, J04.11, J04.2, J05.0, J05.10, J05.11, J04.30, J04.31, J06.0, J06.9, J20.9, J21.0, J21.8, J32.0, J32.1, J32.2, J32.3, J32.4, J32.8, J32.9 | Upper respiratory tract infection |
| 480–488 | J11–J18 | Pneumonia |
| 380.10, 380.11, 380.13, 380.14, 380.16, 381.00, 381.01, 381.02, 381.03, 381.10, 381.19, 381.20, 381.29, 381.3, 381.4, 381.5, 381.51, 381.52, 382.0, 382.00, 382.01, 382.02, 382.3, 382.4, 382.9, 383.00, 383.01, 383.02, 383.1, 384.00, 384.01, 384.09, 384.1 | H65–H68 | Otitis |
| 680–686 | L02–L08, K122, E832 | Cellulitis and abscess |
| 003.21, 032.82, 036.0, 036.1, 036.3, 036.40, 036.41, 036.43, 036.89, 091.81, 100.81, 245.0, 253.8, 254.1, 289.59, 320.0, 320.1, 320.2, 320.3, 320.7, 320.8, 320.81, 320.82, 320.89, 320.9, 322.9, 323.1, 323.4, 323.41, 323.42, 324.0, 324.1, 324.9, 420, 420.0, 420.90, 420.99 | A02.21, A36.81, A39.0, A39.1, A39.81, A39.53, A39.52, A39.89, A51.41, A27.81, E06.0, E23.6, E32.1, D73.3-D73.5, D73.89, G00.0-G00.3, G01, G00.8, G00.9, G04.2, G03.9, G05.3, G05.4, G06.0, G06.1, G06.2, I32, I30.9, I30.8 | Organ infection |
| 003.23, 003.24, 0261, 036.82, 098.50, 098.51, 098.52, 098.53, 098.59, 376.03, 513.1, 519.2, 711.00, 711.01, 711.02, 711.03, 711.04, 711.05, 711.06, 711.07, 711.08, 711.09, 711.90, 711.91, 711.92, 711.93, 711.94, 711.95, 711.96, 711.97, 711.98, 711.99, 730.00, 730.01, 730.02, 730.03, 730.04, 730.05, 730.06, 730.07, 730.08, 730.09, 730.10, 730.11, 730.12, 730.13, 730.14, 730.15, 730.16, 730.17, 730.18, 730.19, 730.20, 730.21, 730.22, 730.23, 730.24, 730.25, 730.26, 730.27, 730.28, 730.29, 730.30, 730.31, 730.32, 730.33, 730.34, 730.35, 730.36, 730.37, 730.38, 730.39, 730.80, 730.81, 730.82, 730.83, 730.84, 730.85, 730.86, 730.87, 730.88, 730.89, 730.90, 730.91, 730.92, 730.93, 730.94, 730.95, 730.96, 730.97, 730.98, 730.99 | A02.23, A02.24, A39.83, A54.42, A54.49, A54.41, A54.40, H05.029, J85.3, J98.51, M00.00, M00.10, M00.20, M00.80, M00.9, M00.019, M00.119, M00.219, M00.819, M00.029, M00.129, M00.229, M00.829, M00.039, M00.139, M00.239, M00.839, M00.049, M00.149, M00.249, M00.849, M00.059, M00.159, M00.259, M00.859, M00.069, M00.169, M00.269, M00.869, M00.079, M00.179, M00.279, M00.879, M00.08,M00.18, M00.28, M00.88, M00.9, M00.09, M00.19, M00.29, M00.89, M01.X0, M01.X19, M01.X29, M01.X39, M01.X49, M01.X59, M01.X69, M01.X79, M01.X8, M01.X9, M86.10, M86.20, M86.119, M86.219, M86.129, M86.229, M86.139, M86.239, M86.149, M86.249, M86.159, M86.259, M86.169, M86.269, M86.179, M86.279, M86.18, M86.28, M86.19, M86.29, M86.60, M86.619, M86.629, M86.639, M86.642, M86.659, M86.669, M86.679, M86.68, M86.69, M86.9, M90.80, M90.819, M90.829, M90.839, M90.849, M90.859, M90.869, M90.879, M90.88, M90.89, M46.30 | Bone infection |
| 998.5, 998.51, 998.59, 996.60, 996.61, 996.62, 996.63, 996.65, 996.66, 996.67, 996.68, 996.69 | K68.11, T85.79XA, T82.6XXA, T82.7XXA, T85.730A, T85.731A, T85.732A, T85.733A, T85.734A, T85.735A, T85.738A, T83.590A, T83.591A, T83.592A, T83.598A, T83.61XA, T83.62XA, T83.69XA, T84.50XA, T84.60XA, T84.7XXA, T85.71XA, T85.79XA | Surgical site infection |
| 020.1, 021.0, 022.0, 032.85, 035, 039.0, 039.3, 039.4, 039.8, 039.9, 040.0, 040.1, 040.2, 040.3, 040.42, 040.81, 078.3, 082.0, 082.1, 082.2, 082.3, 082.40, 082.41, 082.49, 082.8, 082.9, 083.0, 083.1, 083.2, 083.8, 083.9, 087.0, 087.1, 087.9, 088.0, 088.81, 088.82, 088.89, 088.9, 098.50, 567.31, 680.0, 680.1, 680.2, 680.3, 680.4, 680.5, 680.6, 680.7, 680.8, 680.9, 681.00, 681.01, 681.02, 681.10, 681.11, 681.9, 682.0, 682.1, 682.2, 682.3, 682.4, 682.5, 682.6, 682.7, 682.8, 682.9, 683, 684, 685.0, 686.00, 686.01, 686.09, 686.1, 686.8, 686.9, 675.00, 675.01, 675.02, 675.03, 675.04, 675.10, 675.11, 675.12, 675.13, 675.14, 675.80, 675.81, 675.82, 675.83, 675.84, 675.90, 675.91, 675.92, 675.93, 675.94, 705.83, 727.89, 728.0, 728.86 | A20.1, A21.0, A22.0, A36.3, A46, L08.1, A42.2, B47.9, A42.81, A42.82, A42.89, A43.8, A42.9, A43.9, B47.1, A48.0, A48.8, K90.81, A48.52, M60.009, A28.1, A77.0, A77.1, A77.2, A77.3, A77.40, A77.41, A77.49, A77.8, A79.9, A78, A79.0, A79.1, A79.81, A79.89, A79.9, A68.0, A68.1, A68.9, A44.9, A69.20, B60.0, B60.8, B64, A54.42, K68.12, L02.02, L02.03, L02.12, L02.13, L02.221, L02.222, L02.223, L02.224, L02.225, L02.226, L02.229, L02.231, L02.232, L02.233, L02.234, L02.235, L02.236, L02.239, L02.429, L02.439, L02.529, L02.539, L02.33, L02.629, L02.639, L02.821, L02.828, L02.831, L02.838, L02.92, L02.93, L03.019, L03.029, L03.039, L03.049,  K12.2, L03.211, L03.212, L03.213, L03.221, L03.222, L03.319, L03.329, L03.119, L03.129, L03.317, L03.811, L03.818, L03.891, L03.898, L03.90, L03.91, L04.9, L01.00, L01.03, L05.01, L05.02, L05.91, L05.92, L08.0, L88, L08.89, L98.0, E83.2, L08.89, O91.019, O91.011, O91.012, O91.013, O91.02, O91.011, O91.012, O91.013, O91.119, O91.111, O91.112, O91.113, O91.12, O91.23, L73.2, M65.00, M67.80, M67.88, M71.00, M71.80, M60.009, M72.6 | Skin and soft tissue infection |
| 003.1, 020.2, 022.3, 036.2, 036.42, 038.0, 038.1, 038.10, 038.11, 038.12, 038.19, 038.2, 038.3, 038.4, 038.40, 038.41, 038.42, 038.43, 038.44, 038.49, 038.8, 038.9, 040.82, 449, 421.0, 421.1, 421.9, 422.92, 790.7 | A02.1, A20.7, A22.7, A39.4, A39.51, A40.9, A41.2, A41.01, A41.02, A41.1, A40.3, A41.4, A41.50, A41.3, A41.51, A41.52, A41.53, A41.59, A41.89, A41.9, A48.3, I76, I33.0, I39, I33.9, I40.0, R78.81 | Septicemia |
| 995.91, 995.92, 785.52 | A41.9, R65.20, R65.21 | Clinical sepsis |
| 032.0, 032.1, 032.2, 032.3, 032.81, 032.89, 032.9, 033.0, 033.1, 033.8, 033.9, 034.0 | A36.0, A36.1, A36.89, A36.2, A36.86, A36.82, A36.83, A36.84, A36.89, A36.9, A37.00, A37.10, A37.80, A37.90, J02.0, J03.00 | Tonsillitis |
| 487, 488, 008.6, 008.8, 047, 048, 049.0, 049.1, 053.0, 054.72, 321.2, 049.8, 049.9, 052.0, 054.3, 055.0, 056.01, 058.21, 058.29, 061, 062.0, 062.1, 062.2, 062.3, 062.4, 062.5, 066.40, 066.41, 066.42, 066.49, 066.8, 066.9, 053.1, 053.2, 053.7, 053.8, 053.9, 054.0, 054.1, 054.2, 054.4, 054.5, 054.6, 052.1, 052.2, 052.7, 052.8, 052.9, 054.71, 054.73, 054.74, 054.79, 054.8, 054.9, 057, 055.2, 055.7, 055.8, 055.9, 058.8, 056.00, 056.79, 056.8, 056.9, 058.10, 058.11, 074, 079.2, 077, 077.0, 077.1, 077.2, 077.3, 077.4, 077.8, 077.99, 075, 078.5, 079.0, 079.1, 079.6, 466.11, 480.0 | J11.00, J12.9, J10.1, J11.1, J11.2, J11.81, J11.89, J09.X1, J09.X2, J09.X3, J09.X9, J10.08, A08.0, A08.2, A08.11, A08.19, A08.31, A08.32, A08.39, A08.8, A87.0, A87.8, A87.9, A88.0, A87.2, A87.1, B02.1, B00.3, G02, A85.0, A85.1, A85.8, A88.8, A86, A89, B01.11, B00.4, B05.0, B06.01, B10.01, B10.09, A90, A83.0, A83.1, A83.2, A83.3, A83.4, A83.5, A92.30, A92.31, A92.32, A92.39, A93.8, A94, B02.29, B02.39, B02.8, B02.9, B00.0, A60.9, A60.04, B00.2, B00.50, B00.7, B00.89, B00.81, B00.3, B00.1, B00.82, B00.9, B01.2, B01.12, B01.0, B01.81, B01.89, B01.9, B08.3, B09, L44.4, B05.3, B05.81, B05.1, B05.4, B05.89, B05.9, B10.81, B10.82, B10.89, B06.00, B06.81, B06.89, B06.9, B08.20, B08.21, B08.5, B97.11, A74.0, B30.0, B30.2, B30.1, B30.3, B30.8, A74.0, A74.89, B30.9, B27.90, B25.9, B97.0, B97.12, B97.4, J21.0, J12.0 | Viral infections |
| 522.0, 522.5, 522.6, 522.7, 523.00, 523.01, 523.10, 523.11, 523.30, 523.31, 523.32, 523.33, 523.40, 523.41, 523.42, 526.4, 527.3, 528.3, 528.5, 529.0 | K04.01, K04.02, K04.7, K04.5, K04.6, K05.00, K05.01, K05.10, K05.11, K05.20, K05.211,K05.212, K05.213, K05.219, K05.221, K05.222, K05.223, K05.229, K05.30, K05.311,K05.312,K05.313, K05.319, K05.321, K05.322, K05.323, K05.329, M27.2, K11.3, K12.2 C, K13.0, K14.0 | Oral infections |
| 001.0, 001.1, 001.9, 002.0, 002.1, 002.2, 002.3, 002.9, 003.0, 003.20, 003.29, 003.8, 003.9, 004.0, 004.1, 004.2, 004.3, 004.8, 004.9, 005.0, 005.1, 005.2, 005.3, 005.4, 005.8, 005.81, 005.89, 005.9, 008.0, 008.00, 008.01, 008.02, 008.03, 008.04, 008.09, 008.1, 008.2, 008.3, 008.41, 008.42, 008.43, 008.44, 008.46, 008.47, 008.49, 008.5, 009.0, 009.1, 009.2, 009.3, 021.1, 022.2 | A00.0, A00.1, A00.9, A01.00, A01.1, A01.2, A01.3, A01.4, A02.0, A02.20, A02.29, A02.8, A02.9, A03.0, A03.1, A03.2, A03.3, A03.8, A03.9, A04.4, A04.0, A04.1, A04.2, A04.3, A04.8, A04.5, A04.6, A04.9, A05.0, A05.1, A05.2, A05.8, A05.3, A05.5, A05.4, A05.9, , A09, A21.3, A21.2 | Gastroenteritis |
| 091.0, 091.1, 091.2, 091.3, 091.4, 091.50, 091.51, 091.52, 091.61, 091.62, 091.69, 091.7, 091.82, 091.89, 091.9, 092.0, 092.9, 098.0, 098.10, 098.11, 098.12, 098.13, 098.14, 098.15, 098.16, 098.17, 098.19, 098.2, 098.30, 098.31, 098.32, 098.33, 098.34, 098.35, 098.36, 098.37, 098.39, 098.40, 098.41, 098.42, 098.43, 098.49, 098.7, 098.81, 098.82, 098.83, 098.84, 098.85, 098.86, 098.89, 099.0, 099.1, 099.2, 099.3, 099.4, 099.40, 099.41, 099.49, 099.50, 099.51, 099.52, 099.53, 099.54, 099.55, 099.56, 099.59, 099.8, 099.9, 597.80, 597.81, 597.89, 614.0, 614.1, 614.2, 614.3, 614.4, 614.5, 614.7, 614.9, 615.0, 615.1, 615.9, 616.0, 616.10, 616.3, 616.4 | A51.0, A51.1, A51.2, A51.31, A51.32, A51.39, A51.41, A51.43, A51.45, A51.46, A51.49, A51.1, A51.5, A54.00, A54.01, A54.03, A54.21, A54.22, A54.23, A54.24, A54.29, A54.31, A54.32, A54.33, A54.39, A54.6, A54.81, A54.83, A54.85, A54.86, A54.89, A55, A56.00, A56.19, A56.2, A56.3, A56.4, A56.8, A57, A58, A63.8, A64, M02.30, N34.1, N34.2, N34.3, N70.01, N70.02, N70.03, N70.11, N70.12, N70.13, N70.91, N70.92, N70.93, , N73.1, N73.2, N73.3, N73.4, N73.9, N71.0, N71.1, N71.9, N72, N73.0, N76.0, N76.1, N76.2, N76.3 , N75.1, N76.4 | Female pelvic infection |

Abbreviations: ICD-9, International Classification of Disease, 9^th^ Revision; ICD-10, International Classification of Disease,10^th^ Revision; UTI, urinary tract infection.

**S3 Table. Definitions of appropriateness of antibiotic prescriptions [1]**

| **Inappropriate antibiotic prescriptions** | **Suboptimal antibiotic prescriptions** | **Appropriate and optimal antibiotic prescriptions** |
| --- | --- | --- |
| - Prescription of a 2L antibiotic as 1L therapy - Prescription of 2 x 1L antibiotics at the same time - Treatment duration not concurrent with that detailed in IDSA 2011 clinical guidelines | - Treatment failure within 28 days of uUTI diagnosis - Treatment failure defined as:   - Receipt of IV antibiotics   - Switch to a different 1L/2L antibiotic   - Additional primary diagnosis of UTI in an acute care setting (ED or inpatient stay within 28 days of the initial uUTI diagnosis) | - Antibiotic use per IDSA 2011 clinical guidelines for treatment class and duration - 1L FOS, NFT, or  SXT alone - Prescription durations defined as 1 day for FOS, 3 days for FQs and SXT, and 5 days for NFT [1, 2] |

Abbreviations: 1L, first-line; 2L, second-line; ED, emergency department; FOS, fosfomycin; FQ, fluoroquinolone; IDSA, Infectious Diseases Society of America; IV, intravenous; NFT, nitrofurantoin; SXT, trimethoprim‑sulfamethoxazole; UTI, urinary tract infection; uUTI, uncomplicated urinary tract infection.

**S4 Table.** **Associations of HRU and costs (UTI-related and all-cause) for index episode and 12-month follow-up in patients with uUTI, stratified by appropriateness of antibiotic prescription.**

| **Outcome variable^a^** | **Inappropriate/suboptimal antibiotic prescription** | **Appropriate and optimal antibiotic prescription** | **Adjusted difference** |
| --- | --- | --- | --- |
| **Unique patients, n (%)** | 3108 (52.9) | 2762 (47.1) | NA |
| **Index uUTI episode costs, $^b^** | | | |
| Ambulatory care | 582  (575 to 590) | 1430  (1412 to 1449) | 848  (838 to 859) |
| Pharmacy visit | 114  (111 to 116) | 137  (134 to 140) | 23  (23 to 24) |
| **UTI-related costs during 12-month follow-up, $** | | | |
| UTI-related inpatient visit | 39,485  (38,838 to 40,121) | 26,297  (25,866 to  26,721) | -13,188  (-13,400 to  -12,971) |
| UTI-related ED visit | 10,851  (10,686 to 11,024) | 11,717  (11,540 to  11,904) | 867  (853 to 880) |
| UTI-related ambulatory care | 9904  (9718 to 10,093) | 11,932  (11,708 to  12,160) | 2028  (1990 to 2067) |
| UTI-related pharmacy visit | 44  (44 to 44) | 48  (47 to 48) | 4  (4 to 4) |
| **All-cause costs during 12-month follow-up, $** | | | |
| All-cause inpatient visit | 30,874  (30,651 to 31,099) | 29,302  (29,090 to  29,516) | -1572  (-1583 to -1560) |
| All-cause ED visit | 10,081  (9930 to 10,234) | 10,187  (10,034 to  10,341) | 106  (104 to 108) |
| All-cause ambulatory care | 3612  (3577 to 3648) | 3709  (3673 to 3747) | 97  (96 to 98) |
| All-cause pharmacy visit | 1121  (1103 to 1141) | 1208  (1189 to 1229) | 87  (85 to 88) |

Data are adjusted mean (95% CI) unless otherwise stated. All models followed gamma loglink distribution and used recycled prediction modeling with bootstrapping method to estimate 95% CI.

Abbreviations: CI, confidence interval; CCI, Charlson Comorbidity Index; ED, emergency department; HRU, healthcare resource use; NA, not applicable; UTI, urinary tract infection; uUTI, uncomplicated urinary tract infection.

^a^All models were adjusted for age group (reference: 18–39 years), race/ethnicity (reference: White and non-Hispanic), and CCI (reference: 0 CCI).

^b^Inpatient and ED visit costs during the index UTI episode were not modeled because the appropriate and optimal antibiotic prescription group did not incur any costs by definition.

**S5 Table. Treatment sequence for uUTI patients.**

| **First treatment** | | **Second treatment** | | **Third treatment** | |
| --- | --- | --- | --- | --- | --- |
| **Treatment** | **n (%)** | **Treatment** | **n (%)** | **Treatment** | **n (%)** |
| SXT | 1505 (25.6) | SXT + nitrofurantoin | 31 (2.06) |  |  |
|  |  | Nitrofurantoin | 36 (2.39) | Ciprofloxacin | 1 (0.07) |
|  |  |  |  | Levofloxacin | 1 (0.07) |
|  |  | SXT + amoxicillin/clavulanate | 5 (0.33) |  |  |
|  |  | Amoxicillin/clavulanate potassium | 16 (1.06) | Nitrofurantoin | 2 (0.13) |
|  |  | SXT + cefdinir | 2 (0.13) |  |  |
|  |  | Cefdinir | 1 (0.07) |  |  |
|  |  | SXT + ciprofloxacin | 36 (2.39) |  |  |
|  |  | Ciprofloxacin | 45 (2.99) |  |  |
|  |  | SXT + levofloxacin | 7 (0.47) |  |  |
|  |  | Levofloxacin | 5 (0.33) |  |  |
| Nitrofurantoin | 1842 (31.4) | Nitrofurantoin +  SXT | 13 (0.71) |  |  |
|  |  | SXT | 27 (1.47) | Ciprofloxacin | 1 (0.05) |
|  |  | Nitrofurantoin + fosfomycin | 2 (0.11) | SXT | 1 (0.05) |
|  |  | Nitrofurantoin + amoxicillin/clavulanate | 5 (0.27) |  |  |
|  |  | Amoxicillin/clavulanate potassium | 10 (0.54) |  |  |
|  |  | Nitrofurantoin + cefdinir | 1 (0.05) |  |  |
|  |  | Cefdinir | 3 (0.16) |  |  |
|  |  | Nitrofurantoin + ciprofloxacin | 39 (2.12) |  |  |
|  |  | Ciprofloxacin | 48 (2.61) | Amoxicillin/clavulanate | 1 (0.05) |
|  |  |  |  | Levofloxacin | 1 (0.05) |
|  |  |  |  | SXT | 1 (0.05) |
|  |  | Nitrofurantoin + levofloxacin | 4 (0.22) | Cirpfloxacin | 1 (0.05) |
|  |  | Levofloxacin | 9 (0.49) |  |  |
| Amoxicillin/clavulanate | 68 (1.2) | Amoxicillin/clavulanate + SXT | 2 (2.94) |  |  |
|  |  | SXT | 1 (1.47) |  |  |
|  |  | Amoxicillin/clavulanate + nitrofurantoin | 2 (2.94) |  |  |
|  |  | Nitrofurantoin | 1 (1.47) |  |  |
|  |  | Amoxicillin/clavulanate + ciprofloxacin | 2 (2.94) |  |  |
|  |  | Ciprofloxacin | 1 (1.47) |  |  |
|  |  | Amoxicillin/clavulanate + levofloxacin | 1 (1.47) |  |  |
| Cefdinir | 50 (0.9) | Cefdinir + SXT | 1 (2.00) |  |  |
|  |  | SXT | 1 (2.00) |  |  |
|  |  | Cefdinir + nitrofurantoin | 5 (10.00) | Ciprofloxacin | 1 (2.00) |
|  |  | Cefdinir + ciprofloxacin | 1 (2.00) |  |  |
|  |  | Ciprofloxacin | 1 (2.00) |  |  |
|  |  | Levofloxacin | 2 (4.00) | SXT | 1 (2.00) |
| Cefpodoxime proxetil | 3 (0.1) |  |  |  |  |
| Ciprofloxacin | 2266 (38.6) | Ciprofloxacin +  SXT | 28 (1.24) |  |  |
|  |  | SXT | 39 (1.72) | Amoxicillin/clavulanate | 1 (0.04) |
|  |  |  |  | Levofloxacin | 1 (0.04) |
|  |  |  |  | Nitrofurantoin | 3 (0.13) |
|  |  | Ciprofloxacin + nitrofurantoin | 46 (2.03) |  |  |
|  |  | Nitrofurantoin | 46 (2.03) |  |  |
|  |  | Ciprofloxacin + amoxicillin/clavulanate | 7 (0.31) |  |  |
|  |  | Amoxicillin/clavulanate | 12 (0.53) |  |  |
|  |  | Ciprofloxacin + cefdinir | 2 (0.09) |  |  |
|  |  | Cefdinir | 4 (0.18) | Amoxicillin/clavulanate | 1 (0.04) |
|  |  | Ciprofloxacin + levofloxacin | 1 (0.04) |  |  |
|  |  | Levofloxacin | 9 (0.40) |  |  |
| Levofloxacin | 136 (2.3) | Levofloxacin +  SXT | 1 (0.74) |  |  |
|  |  | SXT | 0 (0.00) |  |  |
|  |  | Nitrofurantoin | 2 (1.47) |  |  |
|  |  | Amoxicillin/clavulanate | 2 (1.47) |  |  |
|  |  | Levofloxacin + ciprofloxacin | 1 (0.74) |  |  |
|  |  | Ciprofloxacin | 1 (0.74) |  |  |

Antibiotics were considered overlapping if the next antibiotic was filled before the completion date of the first antibiotic prescription, and subsequent if the next antibiotic was filled after the completion date of the first antibiotic prescription.

Abbreviations: SXT, trimethoprim sulfamethoxazole; uUTI, uncomplicated urinary tract infection.

# References

- 1. Gupta K, Hooton TM, Naber KG, Wullt B, Colgan R, Miller LG, et al. International clinical practice guidelines for the treatment of acute uncomplicated cystitis and pyelonephritis in women: A 2010 update by the Infectious Diseases Society of America and the European Society for Microbiology and Infectious Diseases. Clin Infect Dis. 2011;52(5):e103-20. Epub 2011/02/05. doi: 10.1093/cid/ciq257. PubMed PMID: 21292654.
- 2. Robinson D, Giarenis I, Cardozo L. The management of urinary tract infections in octogenarian women. Maturitas. 2015;81(3):343-7. Epub 2015/05/27. doi: 10.1016/j.maturitas.2015.04.014. PubMed PMID: 26006302.
